# AstigMETRICS: An Automated Tool for Standardized Vector Metrics Tables and Group Comparisons in Refractive Surgery

**DOI:** 10.3390/jcm15052018

**Published:** 2026-03-06

**Authors:** Mathieu Gauvin, Avi Wallerstein

**Affiliations:** 1 Department of Ophthalmology and Visual Sciences, McGill University, Montreal, QC H4A 3S5, Canada; mathieu.gauvin@mcgill.ca; 2 LASIK MD, Montreal, QC H4A 3S5, Canada

**Keywords:** astigmatism vector analysis, target-induced astigmatism, surgically induced astigmatism, difference vector, correction index, magnitude of error, angle of error, index of success, refractive surgery outcomes, standardized reporting

## Abstract

**Background/Objectives**: Standardized reporting of astigmatism outcomes is essential for comparability, reproducibility, and interpretation of refractive surgery studies. Vectorial analyses based on established metrics are increasingly required by major journals, yet no freely available tool exists for generating publication-ready vector analysis tables with statistical comparisons. This study presents AstigMETRICS, a standalone application for automated calculation, formatting, and statistical comparison of standard vector metrics in refractive surgery. **Methods**: AstigMETRICS was developed in MATLAB and compiled as a standalone executable requiring no programming knowledge. The software accepts preoperative, intended, and postoperative astigmatism data in spreadsheet format for both refractive and corneal measurements. It calculates seven standard vector metrics following the Alpins method: the target-induced astigmatism (TIA), surgically induced astigmatism (SIA), difference vector (DV), correction index (CI), magnitude of error (ME), angle of error (AE), and index of success (IOS). Statistical comparisons are performed automatically using appropriate parametric or nonparametric tests for paired and unpaired study designs, with *p*-values and Cohen’s d effect sizes reported. **Results**: AstigMETRICS generates standardized tables reporting the means, standard deviations, and clinically relevant proportions (percentage of eyes with an ME within ±0.50 D or ±1.00 D, and an AE within ±15°). Three simulated datasets were created to validate the software functionality across common surgical scenarios: a contralateral eye laser vision correction, toric phakic IOL implantation, and cataract surgery with toric IOLs. The output tables are displayed in standardized format and saved as high-resolution TIFF images suitable for publication. The software is freely available and a download link is provided in this article. **Conclusions**: AstigMETRICS enables clinicians and researchers to perform standardized, reproducible astigmatism vector analyses with built-in statistical comparisons. This freely available tool simplifies outcome reporting and improves methodological consistency in refractive surgery research.

## 1. Introduction

Standardized reporting of surgical outcomes enhances reproducibility and enables meaningful comparisons across clinical studies [1,2]. In refractive surgery, outcome reporting standards were first introduced by Waring in 1992 [3] and later formalized into six figures summarizing the accuracy, efficacy, safety, and stability [4,5,6,7,8]. These figures are now required in manuscripts submitted to leading journals, including the *Journal of Refractive Surgery* (JRS) [6], the *Journal of Cataract and Refractive Surgery* (JCRS) [6], *Cornea* [6], and *AAO Ophthalmology* [8]. Adhering to these specifications allows for outcomes from original studies, case series, and clinical trials to be reported consistently and compared reliably within and between refractive surgery studies [1].

Beyond these six figures, major refractive surgery journals such as JRS [1] and JCRS [9] also require vector analyses [8,10,11,12,13], especially when astigmatism correction is a central outcome. Vector analyses provide a structured framework for quantifying the effectiveness of astigmatic correction, with each vector metric addressing distinct clinical questions. Vector analyses are applicable to any ocular procedure where astigmatism correction needs to be rigorously evaluated, including corneal-based surgeries such as PRK, LASIK, LASEK, SMILE, incisional keratotomy, collagen cross-linking, and intracorneal ring segments. It is equally relevant for lens-based procedures such as phakic IOL implantation, cataract surgery, and refractive lens exchange with multifocal and/or toric IOLs. Vector methods are also valuable for assessing modified surgical techniques and interventions used to manage complications in refractive surgery [14,15,16,17,18,19,20,21,22,23].

While graphical representations of astigmatism outcomes are increasingly required, many studies complement them with vector analysis tables reporting key metrics, including the means and standard deviations of the target-induced astigmatism (TIA), surgically induced astigmatism (SIA), difference vector (DV), correction index (CI), index of success (IOS), magnitude of error (ME), and angle of error (AE), as well as the proportion of eyes achieving an ME within ±0.50 D or ±1.00 D, and the proportion of eyes with an AE within ±15 degrees, an AE >15 degrees, and an AE < −15 degrees. These numerical outcomes are equally essential for rigorous research, standardized reporting, and meaningful statistical comparisons across studies.

Despite their importance, no freely available or commercial software currently provides a standardized solution for generating these vector analysis tables with automated statistical comparisons between groups. To address this gap, we developed AstigMETRICS, a free standalone executable application that calculates the full set of vector analysis metrics and produces standardized publication-ready tables from manifest or corneal data. Unlike existing solutions, AstigMETRICS also performs automated group comparisons using appropriate hypothesis testing for both paired and unpaired study designs. This tool is designed to assist clinicians and researchers in evaluating astigmatism outcomes across surgical techniques and to streamline the integration of robust, publication-ready vector metrics tables into scientific manuscripts, abstracts, and presentations.

## 2. Materials and Methods

### 2.1. Standard Metrics Reported by AstigMETRICS

The nomenclature and metrics introduced by Alpins [8,10,11,12,13] have become the prevailing standard for vectorial analyses of astigmatism in refractive surgery, with widespread adoption across hundreds of peer-reviewed publications over the past 30 years. Leading journals such as JRS [1] and JCRS [9] endorse this approach for studies in which astigmatism correction is a primary endpoint. The Alpins method defines and quantifies astigmatism correction using well-established parameters, including:

1. Target-induced astigmatism (TIA): the intended astigmatic change defined by preoperative planning.
2. Surgically induced astigmatism (SIA): the astigmatic effect actually achieved by the procedure.
3. Difference vector (DV): the residual astigmatism error, representing the vectorial discrepancy between the TIA and the SIA.
4. Correction index (CI): the ratio of the SIA to the TIA, used to assess undercorrection (CI < 1) or overcorrection (CI > 1), with an ideal value of 1.
5. Index of success (IOS): the ratio of the DV to the TIA, providing a normalized measure of the residual error relative to the intended target, with a smaller value indicating a better outcome.
6. Magnitude of error (ME): the arithmetic difference in magnitude between the SIA and the TIA, indicating the extent of undercorrection (if negative) or overcorrection (if positive).
7. Angle of error (AE): the angular difference between the intended and achieved vector axes, used to assess clockwise and counterclockwise rotational misalignments.

AstigMETRICS is built upon the above standardized nomenclature and provides fully automated standard tables of these seven metrics, including group means or geometric means, standard deviations, and clinically relevant proportions of eyes achieving an ME within ±0.50 D and ±1.00 D, and proportions of eyes achieving an AE within ±15°, AE < 15 and AE > 15. While some reports have referenced the American National Standards Institute (ANSI) vector terminology [24], its use remains uncommon and is discouraged in favor of the Alpins methods. AstigMETRICS uses the Alpins’ nomenclature, as it remains the most widely used terminology [8,10,11,12,13].

### 2.2. Software Implementation and System Requirements

The software was developed in MATLAB R2024b (Mathworks Inc., Natick, MA, USA) and compiled into a standalone executable format. AstigMETRICS operates independently of MATLAB installations and licenses, requiring only the pre-installation of the associated MATLAB runtime compiler (MRC) on the user’s system. It operates as a fully local desktop application, ensuring data privacy and security by keeping all user data on the local machine. AstigMETRICS has been tested on Windows 10 and 11 (Home and Professional), with a 64-bit operating system, and with a screen resolution of 1920 × 1080, or 3840 × 2160, and 1920 × 2400. AstigMETRICS and the demonstration datasets are available to download from: http://www.lasikmd.com/media/astigmetrics (accessed on 7 December 2025).

### 2.3. Input Data Format

To automatically generate a standard vector analysis table, AstigMETRICS reads data files in Microsoft Excel format (e.g., Datafile.xlsx). Excel was chosen due to its widespread use and simplicity. A total of 12 columns are mandatory for proper functioning of the software. The first four columns are: (1) the preoperative sphere, (2) the preoperative cylinder/astigmatism magnitude, (3) the preoperative cylinder/astigmatism axis (in degrees), and (4) the preoperative vertex distance (in millimeters). The next four columns are: (5) the intended postoperative target sphere, (6) the intended postoperative target cylinder/astigmatism magnitude, (7) the intended postoperative target axis (in degrees), and (8) the intended vertex distance. If the intended postoperative refraction is plano, columns 5, 6, 7, and 8 should be reported as 0, 0, 0, and 12, respectively. The final four columns are: (9) postoperative sphere, (10) postoperative cylinder/astigmatism magnitude, (11) postoperative cylinder axis (in degrees), and (12) postoperative vertex distance. Refraction values must be reported in decimal notation (e.g., −1.50, 0.75) using the negative cylinder format (-ve), which remains the most used convention among refractive surgeons. AstigMETRICS automatically converts values to positive cylinder notation for calculation purposes. AstigMETRICS also supports the evaluation of corneal astigmatism vector changes, such as those derived from topography or keratometry. In such cases, users may input corneal astigmatism values directly into the cylinder and axis fields and enter 0 for all sphere and vertex distance columns. This approach allows for flexible analysis of either refractive or corneal plane vector outcomes within the same input structure. As per standard practice in astigmatism vector analyses, AstigMETRICS automatically excludes eyes with a target-induced astigmatism (TIA) of less than 0.25 D at the corneal plane. Additionally, any row containing missing data in one or more of the required 12 columns is automatically excluded from the analysis. For paired-group comparisons, if corresponding rows in the two data files contain missing data, both rows are excluded from the analysis. Users should ensure data completeness before the analysis.

### 2.4. Program Workflow

**Figure 1:**
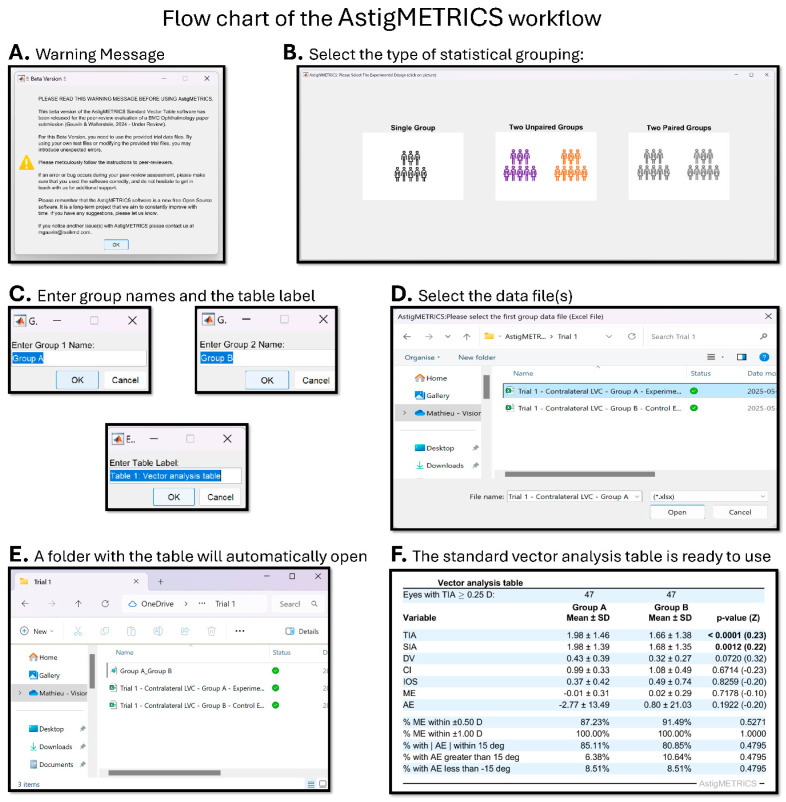
Flow chart of the AstigMETRICS workflow. Upon starting the application, the user is warned about data formatting (**A**) and invited to select the type of statistical grouping (**B**). The user is then invited to enter the group name(s) and the label for the standard vector table (**C**). The user is next invited to select the data file(s) (**D**). The table is automatically displayed and saved as high-resolution TIFF images in a folder at the same location as the original Excel data file (**E**). This folder automatically opens once the graphs are saved. The user can visualize the generated standard table (**F**).

The flow chart of the AstigMETRICS workflow is shown in Figure 1. The application was designed to be user-friendly and accessible to users without any knowledge of MATLAB coding. Upon launching, the user is first presented with a warning dialog that summarizes the input data requirements (Figure 1A), followed by a prompt to select the type of statistical grouping: single-group, two-group paired, or two-group unpaired (Figure 1B). The user is then asked to enter the group name(s) and a label for the standard vector analysis table (Figure 1C). Next, the appropriate Excel data file(s) must be selected; one file for a single-group analysis or two files for two-group comparisons (Figure 1D). AstigMETRICS automatically processes the input, generates the standard vector analysis table, and saves it as a high-resolution TIFF image in the same directory as the original data file. This folder automatically opens upon saving (Figure 1E). The formatted table is also immediately displayed for user review (Figure 1F). 

### 2.5. Calculation of Astigmatism Vectors

Astigmatism vectorial analyses were performed using the established Alpins method for calculating the four fundamental stigmatism vectors: the target-induced astigmatism (TIA), surgically induced astigmatism (SIA), difference vector (DV), and correction index (CI). The computational methodology, including coordinate system conversions and trigonometric transformations, follows standard protocols as comprehensively detailed in prior publications [8,10,11,12,13]. In addition to these classical vectors, AstigMETRICS computes the magnitude of error (ME), defined as the absolute scalar difference between the TIA and SIA, and the angle of error (AE), defined as the angular difference between these two vectors. The index of success (IOS), calculated as the DV divided by the TIA, is also reported as a measure of residual astigmatism relative to the target. AstigMETRICS further outputs clinically relevant proportions of eyes achieving an ME within ±0.50 D or within ±1.00 D, and an AE within ±15°, greater than 15°, or less than −15°. Interested readers seeking detailed mathematical derivations and calculation procedures should refer to the foundational literature [8,10,11,12,13].

### 2.6. Data and Statistical Analysis

To support group comparisons, AstigMETRICS conducts hypothesis testing on all vector-derived numerical metrics. For each variable, normality is assessed using a one-sample Kolmogorov–Smirnov goodness-of-fit test against a standard normal distribution. If both groups meet the assumption of normality (*p* > 0.05), AstigMETRICS applies parametric tests: paired *t*-tests for within-subject comparisons and independent two-sample *t*-tests for unpaired designs. When the normality assumption is not met in either or both groups, nonparametric alternatives are used: the Wilcoxon signed-rank test for paired data and the Wilcoxon rank-sum test (equivalent to the Mann–Whitney U test) for unpaired data. For binary metrics based on clinical thresholds, such as the proportion of eyes with a magnitude of error (ME) within ±0.50 D, within ±1.00 D, or with an angle of error (AE) within 15 degrees, AstigMETRICS applies a chi-square test to unpaired comparisons and McNemar’s test to paired data. While McNemar’s test is appropriate for before-and-after measurements on the same eyes, it may not fully address the within-subject correlation in contralateral eye designs, and additional modeling may be required for such analyses. All *p*-values are reported to four decimal places to enhance the reproducibility and transparency. In addition, Cohen’s d effect sizes were calculated and are reported to two decimal places for each variable. This integrated statistical framework allows AstigMETRICS to support both paired and unpaired study designs, ensuring that the resulting outputs are statistically valid, clinically interpretable, and ready for inclusion in peer-reviewed publications. For greater statistical validity, in unpaired or single-group analyses, the user should include the outcome from one eye per patient in the input data file, such as the dominant eye or a randomly selected eye.

## 3. Results

**Figure 2:**
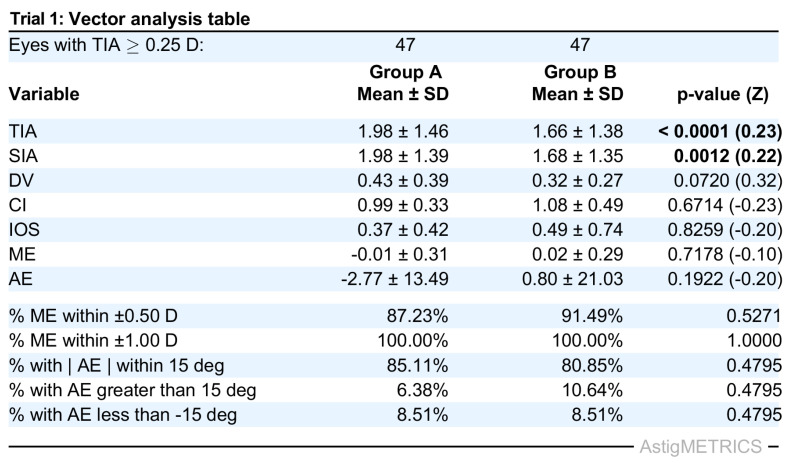
Standard table that is automatically generated by AstigMETRICS from the provided Trial 1 dataset. The first simulated trial dataset (Trial 1) includes two Excel files (Group A and Group B) and investigates the outcomes of a laser vision correction contralateral eye study comparing two excimer lasers in hyperopic eyes with astigmatism.

**Figure 3:**
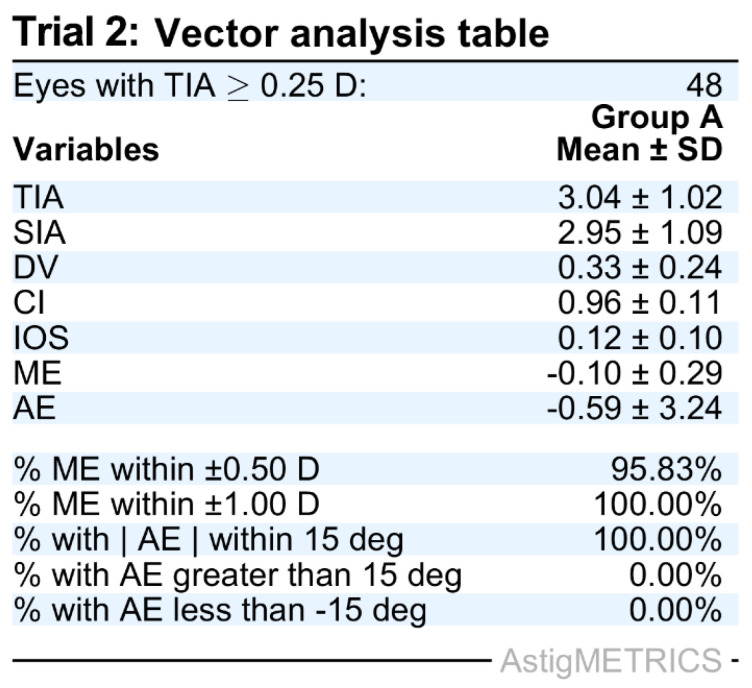
Standard table that is automatically generated by AstigMETRICS from the provided Trial 2 dataset. The second dataset (Trial 2) comprises simulated data from a single group in order to investigate the outcomes of a toric phakic IOL (PIOL) in hyperopic eyes with moderate to high astigmatism.

**Figure 4:**
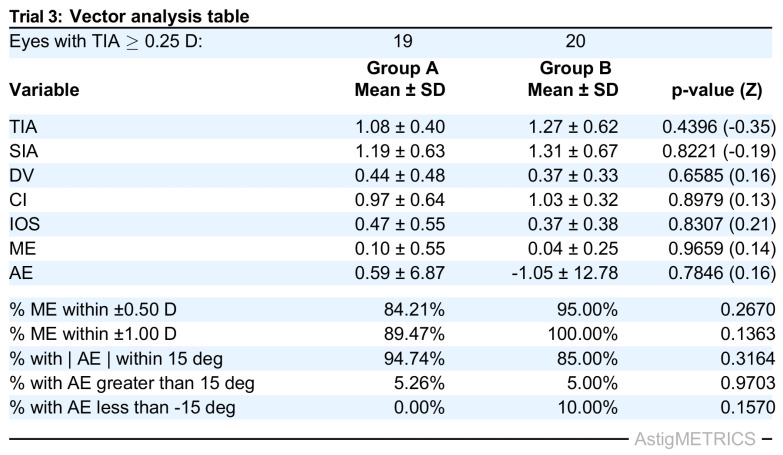
Standard table that is automatically generated by AstigMETRICS from the provided Trial 3 dataset. The third simulated dataset (Trial 3) includes two files (Group A and Group B), used to investigate the outcomes of two cataract surgery groups, comparing two biometers, in myopic-astigmatism eyes.

To validate the functionality and demonstrate the capabilities of AstigMETRICS, we produced three simulated datasets reflecting common refractive and lens-based surgical scenarios. These datasets are freely available for users to test the software and reproduce the example outputs. The first simulated dataset (Trial 1) includes two Excel files (Group A and Group B) designed to mimic a contralateral eye laser vision correction study comparing two excimer laser platforms in hyperopic eyes with astigmatism. The second dataset (Trial 2) simulates a single-group study evaluating toric phakic intraocular lens (PIOL) implantation in hyperopic eyes with moderate to high astigmatism. The third dataset (Trial 3) comprises two groups and simulates a cataract surgery study comparing two biometry devices in myopic eyes with astigmatism. In each case, AstigMETRICS reads the input Excel files, performs the full set of astigmatism vector calculations, and automatically generates the standardized vector analysis table. As shown in Figure 2, Figure 3 and Figure 4, these tables include mean ± standard deviation values for each metric, proportions of eyes meeting clinically relevant thresholds, and the corresponding *p*-values and effect sizes for group comparisons. The tables are automatically formatted for publication and saved as high-resolution TIFF files. The calculated vector averages (e.g., TIA, SIA, DV, CI) match those reported in our previously published mEYEstro software paper, thereby validating the accuracy and internal consistency of AstigMETRICS. Unlike graphical tools such as mEYEstro or AstigMATIC, AstigMETRICS focuses exclusively on producing publication-ready vector analysis tables formatted for journal submission, abstract reporting, or internal quality assurance. Users are encouraged to first test the software using the three included trial datasets, available at: http://www.lasikmd.com/media/astigmetrics (accessed on 7 December 2025). These examples cover single-group, two-group paired, and two-group unpaired scenarios. Once familiar with the workflow, users can simply overwrite the content of any trial file with their own data to perform customized analyses while preserving the required input format.

## 4. Discussion

AstigMETRICS can be used to automatically generate standardized vector analysis tables for astigmatism correction outcomes, based on the Alpins method, as recommended by leading refractive surgery and ophthalmology journals [8,10,11,12,13]. Originally developed for academic research and teaching in the context of laser refractive surgery, the tool is also applicable to the evaluation of astigmatism outcomes following corneal or intraocular procedures, including phakic and pseudophakic lens surgery outcomes [14,15,16,17,18,19,20,21,22,23]. Users are requested to cite the present manuscript in any publication, presentation, or public communication, whether peer-reviewed or not, when reporting results generated using AstigMETRICS. For comprehensive and standardized refractive surgery outcome reporting, we recommend pairing AstigMETRICS with AstigMATIC [25] (for standard vector plots) and mEYEstro [26] (for accuracy, efficacy, safety, and stability standard graphs).

### 4.1. Limitations

The primary advantage of AstigMETRICS lies in its ability to automate the generation of standardized vector analysis tables and statistical comparisons through a user-friendly graphical interface, without requiring any programming expertise or knowledge of MATLAB. However, this ease of use comes with inherent limitations. As a compiled standalone application rather than an editable MATLAB script, AstigMETRICS offers limited flexibility for users seeking customization. Modifying visual elements such as fonts, colors, or table formatting is not supported. This constraint was intentional, as the formatting was standardized to comply with current journal recommendations [1,9], thereby promoting consistency and facilitating cross-study comparisons. Additionally, AstigMETRICS is specifically designed for astigmatism vector analyses and does not support calculations related to other refractive errors, such as the spherical equivalent.

### 4.2. Improvements and Future Work

AstigMETRICS was developed to address the absence of standardized, automated tools for generating vector analysis tables in refractive surgery. The current version includes built-in statistical testing for both paired and unpaired comparisons, and reports *p*-values (to four decimals) and Cohen’s d effect sizes (to two decimals) for all supported metrics. However, the software is currently limited to single- or two-group comparisons. Future versions may incorporate support for more complex study designs, including three- or multi-group comparisons using advanced statistical methods such as an analysis of variance (ANOVA) and post hoc testing. Additional developments could include the integration of alternative astigmatism analysis frameworks, such as ANSI vector notation, based on user demand, as well as the incorporation of new metrics as they emerge in the refractive surgery literature. A cloud-based version of the software may also be considered to enable real-time, browser-based access for clinicians and researchers across multiple sites, though such development would depend on the scale of user adoption and community feedback. As with all vector-based outcome analyses, users are advised to include only one eye per patient, such as the dominant or a randomly selected eye, to avoid inter-eye correlation biases, unless an appropriate paired-eye analysis is conducted.

### 4.3. Significance of the AstigMETRICS Software

Astigmatism correction is inherently multidimensional, requiring evaluation of both the magnitude and axis [8,10,11,12,13]. While graphical vector representations provide valuable insight into the surgical accuracy, quantitative reporting of key metrics remains essential for evaluating the outcomes, identifying sources of residual astigmatism, and comparing treatment strategies. Despite the importance of these metrics, no free, standardized solution previously existed for the automated calculation and reporting of astigmatism vector outcomes. AstigMETRICS addresses this gap by providing researchers and clinicians with an accessible, standardized platform that applies recognized vector analysis methods [8,10,11,12,13]. In addition to calculating the full set of vector metrics, the software supports group comparison capabilities and hypothesis testing adapted for both paired and unpaired study designs. By streamlining the generation of high-quality, reproducible, publication-ready tables and incorporating statistical evaluations, AstigMETRICS facilitates the inclusion of advanced astigmatism analyses in clinical research and outcome reporting as per the latest requirements of JRS [1] and JCRS [9]. For comprehensive and standardized refractive surgery outcome reporting, we recommend pairing AstigMETRICS with AstigMATIC [25] (for standard vector plots) and mEYEstro [26] (for accuracy, efficacy, safety, and stability standard graphs).

## 5. Conclusions

AstigMETRICS is a freely available software tool designed to support clinicians and researchers in the standardized reporting of astigmatism treatment outcomes. It enables the rapid generation of publication-ready vector analysis tables, complete with key outcome metrics and statistical comparisons, thereby facilitating rigorous and reproducible reporting for scientific manuscripts, academic presentations, research studies, and clinical quality assurance and knowledge.

## Data Availability

All data generated or analyzed during the current study are included in this published article and are available in the data repository at http://www.lasikmd.com/media/astigmetrics (accessed on 7 December 2025).

## Abbreviations

The following abbreviations are used in this manuscript:

AE	Angle of Error
CI	Correction Index
DV	Difference Vector
IOL	Intraocular Lens
IOS	Index of Success
JCRS	*Journal of Cataract and Refractive Surgery*
JRS	*Journal of Refractive Surgery*
LASEK	Laser Epithelial Keratomileusis
LASIK	Laser-Assisted In Situ Keratomileusis
ME	Magnitude of Error
MRC	MATLAB Runtime Compiler
PIOL	Phakic Intraocular Lens
PRK	Photorefractive Keratectomy
SIA	Surgically Induced Astigmatism
SMILE	Small Incision Lenticule Extraction
TIA	Target-Induced Astigmatism
